# Penta-o-galloyl-beta-d-Glucose (PGG) inhibits inflammation in human rheumatoid arthritis synovial fibroblasts and rat adjuvant-induced arthritis model

**DOI:** 10.3389/fimmu.2022.928436

**Published:** 2022-08-10

**Authors:** Sadiq Umar, Anil K. Singh, Mukesh Chourasia, Stephanie M. Rasmussen, Jeffrey H. Ruth, Salahuddin Ahmed

**Affiliations:** ^1^ Department of Pharmaceutical Sciences, Washington State University College of Pharmacy and Pharmaceutical Sciences, Spokane, WA, United States; ^2^ Center for Computational Biology and Bioinformatics, Amity Institute of Biotechnology, Amity University Uttar Pradesh, Noida, India; ^3^ Division of Rheumatology, University of Michigan Medical School, Ann Arbor, MI, United States; ^4^ Division of Rheumatology, University of Washington School of Medicine, Seattle, WA, United States

**Keywords:** rheumatoid arthritis, synovial fibroblasts, post-translational modification, O-GlcNAcylation, TGF beta-activated kinase 1 (TAK1), Penta-o-galloyl-beta-D-glucose (pgg)

## Abstract

O-GlcNAcylation is a reversible post-translational modification that regulates numerous cellular processes, including embryonic development as well as immune responses. However, its role in inflammation remains ambiguous. This study was designed to examine the role of O-GlcNAcylation in rheumatoid arthritis (RA) and its regulation using human RA patient-derived synovial fibroblasts (RASFs). The efficacy of penta-O-galloyl-beta-D-glucose (PGG), a potent anti-inflammatory molecule, in regulating inflammatory processes in human RASFs was also evaluated. Human synovial tissues and RASFs exhibited higher expression of O-GlcNAcylation compared to their non-diseased counterparts. Pretreatment of RASFs with Thiamet G, an inhibitor of O-GlcNAcase, markedly increased the O-GlcNAc-modified proteins and concomitantly inhibited the IL-1β-induced IL-6 and IL-8 production in human RASFs *in vitro.* Pretreatment of human RASFs with PGG (0.5-10 µM) abrogated IL-1β-induced IL-6 and IL-8 production in a dose-dependent manner. Immunoprecipitation analysis showed that PGG inhibited O-GlcNAcylation of TAB1 to reduce its association with TGF β-activated kinase 1 (TAK1) and its autophosphorylation, an essential signaling step in IL-1β-induced signaling pathways. Molecular docking *in silico* studies shows that PGG occupies the C174 position, an ATP-binding site in the kinase domain to inhibit TAK1 kinase activity. Oral administration of PGG (25 mg/kg/day) for 10 days from disease onset significantly ameliorated rat adjuvant-induced (AIA) in rats. PGG treatment reduced the phosphorylation of TAK1 in the treated joints compared to AIA joints, which correlated with the reduced disease severity and suppressed levels of serum IL-1β, GM-CSF, TNF-α, and RANKL. These findings suggest O-GlcNAcylation as a potential therapeutic target and provide the rationale for testing PGG or structurally similar molecule for their therapeutic efficacy.

## Introduction

Post-translational modifications (PTMs) serve an important role in regulating signal-transduction pathways by alteration of existing protein and modulation of its function. It includes phosphorylation, acetylation, glycosylation, ubiquitination, and hydroxylation (1–3). Phosphorylation and ubiquitination are extensively studied PTMs, however, it has become increasingly clear that numerous other covalent changes transpire to existing proteins (4–6). Among these important regulatory mechanisms, glycosylation, an addition of the single-sugar N-acetylglucosamine (O-GlcNAc) to serine or threonine on cytosolic and nuclear proteins are gaining significant relevance in various chronic and age-related diseases (7, 8). Recent studies showed that interleukin-1β (IL-1β), a key catabolic cytokine can induce O-GlcNAc accumulation in osteoarthritis (9–11). In addition, glycosylation of the IL-1 receptor (IL-1R) is required for the optimal binding and signaling of IL-1β (12). However, its role in rheumatoid arthritis (RA) remains unexplored.

IL-1β is unquestionably the most broadly studied cytokine of the IL-1 family and is involved in a wide range of inflammatory diseases, including RA, osteoarthritis (OA), gout, periodic fever, and type II diabetes (13). IL-1 comprises IL-1α and IL-1β that are produced by various different cells, including macrophages, chondrocytes, osteoblasts, and synoviocytes. The signal initiation occurs by attachment of IL-1 to the IL-1R complex which leads to the recruitment of MyD88, that in turn recruits IL-1 receptor-associated kinases (IRAKs) (14). TRAF6, an E3 ubiquitin ligase, forms a complex with IRAK-1 that dissociates from the receptor (15) and translocates to the cytoplasm to recruit and activate TAK1 at its kinase domain threonine (Thr^184/187^), resulting in the recruitment of downstream signaling molecules and activation of the nuclear factor kappa B (NF-κB) and MAPK (p38, JNK, and ERK) pathways (16). Therefore, identifying targets to validate new agents that can effectively interfere with IL-1β-activated signaling pathways may have therapeutic value in RA as well as other IL-1β-driven inflammatory diseases.

In this regard, our lab showed that an anti-inflammatory compound epigallocatechin-3 gallate (EGCG) selectively inhibits TAK1 by blocking its phosphorylation and hindering association with TRAF6 through down-regulation of TRAF6-associated K^63^-linked autoubiquitination (17). Other studies highlight the activation of TNFAIP3 (A20) associated with inflammatory disorders (18), as a mechanism for negative regulation of NF-κB by its deubiquitinase activity, while Igarashi et al. showed its proinflammatory activity (19). A20 may exert this suppressive effect on activated NF-κB through the regulation of ubiquitin status of signaling molecules with its dual enzymatic activities; ubiquitination and deubiquitination (20). In addition, NF-κB is a pleiotropic transcription factor that significantly upregulates the expression of pro-inflammatory cytokines (TNF-α, IL-1β, IL-6, and IL-17) in the synovial joint resulting in the increased expression of RANKL (receptor activator of nuclear factor-kB ligand), which in turn stimulates macrophages to participate in osteoclastogenesis resulting in bone destruction in RA (21, 22).

1,2,3,4,6-Penta-O-galloyl-beta-D-glucose (PGG) is a plant-derived compound found to possess biological activities, including anti-proliferative, anti-angiogenic, apoptotic, and anti-diabetic activities (23–28). The present study was carried out to understand the anti-inflammatory effects of PGG in human RA synovial fibroblasts (RASFs) and a rat adjuvant-induced arthritis (AIA) model of human RA.

## Materials and methods

### Antibodies and reagents

Recombinant human IL-1β, IL-6, IL-8, RANTES, and MMP-1 ELISA assays were purchased from R&D Systems (Minneapolis, MN). PGG was purchased from Sigma (G7548 >95% pure) for *in vitro* and from Cayman Chemicals (#16007) for animal study. Thiamet G (#13237, Cayman Chemicals), human cytokine antibody array C5 (AAH-CYT-5-2) and TRANCE ELISA (ELM-TRANCE-1) from RayBiotech (Norcross, GA), rat Cytokine/Chemokine Magnetic Bead Panel (RECYMAG65K27PMX; Sigma, MO), mouse O-GlcNAc (CTD110.6) (sc-59623), mouse A20 (#sc-166692), and mouse monoclonal β-actin (sc-47778) antibodies were purchased from Santa Cruz Biotech (Santa Cruz, CA). Antibodies against p-TAK1 Thr^184/187^ (#4531), p-JNK (#9251) p-JNK (#4511), p-c-Jun (Ser^73^) (#9164), MyD88 (D80F5) (#4283), CYLD (D6O5O) (#12797) were purchased from Cell Signaling Technology (Beverly, MA). Anti-TAK1 (ab109526) and anti-TRAF6 (ab33915) antibodies were from Abcam (Cambridge, MA). Goat anti-rabbit and goat anti-mouse HRP-linked secondary antibodies were purchased from Cell Signaling Technology.

### Culture of human RASFs

Human SFs were isolated from RA synovium obtained according to the Institutional Review Board (IRB) approved protocol in compliance with the Helsinki Declaration from patients who had undergone total joint replacement surgery or synovectomy and processed as described previously (29).

### Human cytokine array

In the first set of experiments, we sought to identify RASF cytokines that were modulated by PGG treatment. Human RASFs (2 x 10^5^/well) were plated in 60 mm dishes and pretreated with or without PGG (5 μM) for 2 hours followed by IL-1β (10 ng/ml) stimulation for 24 hours. Conditioned media collected was used in Human Cytokine Antibody Array (AAH-CYT-5-2) to analyze 80 human cytokines as per kit recommendation.

### Treatment of human RASFs

To evaluate the time-dependent activation of IL-1β-induced signaling pathways and the protective effect of PGG treatment, RASFs (2 x 10^5^/well) were plated in 6-well plates with or without PGG (0.5-10 μM) pretreatment for 2 hours followed by IL-1β (10 ng/ml) stimulation for 30 minutes (for signaling studies) or 24 hours to evaluate the production of IL-6, IL-8, MMP-1 and RANTES in the conditioned media. To understand the mechanism of O-GlcNAc in IL-β signaling, RASFs were incubated with the inhibitor of enzyme O-GlcNAcase (Thiamet G; 1-5 μM), overnight followed by stimulation with IL-1β for 24 hours to evaluate the production of IL-6 and IL-8 in the conditioned media or cellular expression of O-GlcNAc.

### TAK1 *in vitro* kinase activity

*In vitro* TAK1 kinase assay was performed according to the manufacturer’s instructions (Promega, Madison, WI; catalog V4088). The concentration range of 0.01-5 μM was used for validation of PGG’s inhibitory properties. The experiment was carried out in 96- well plate format and 25 μl total reaction volume. Different concentrations of PGG were incubated with TAK1-TAB1 fusion protein and substrate mix for 10 minutes. ATP (500 μM) was then added to initiate kinase activity followed by 60 minutes of incubation at room temperature. After 60 minutes, 25 μl of ADP-Glo was added and incubated for an additional 40 minutes at room temperature. Upon 40 minutes incubation reaction mixture was incubated with 50 μl of kinase detection reagent. Luciferase activity was recorded after 30 minutes.

### Western blotting analysis

Western blot analysis was performed as described earlier (29, 30). To study the effects of IL-1β, whole-cell extracts were prepared using RIPA buffer (50 mM Tris pH 7.6, 150 mM NaCl, 1% Triton X100, 1mM EDTA, 0.5% sodium deoxycholate, 0.1% SDS) containing protease inhibitor and phospho-Stop tablets (Roche, Indianapolis, IN). The joint homogenates were prepared using ankles from the rat AIA study as described earlier (17). Protein was measured using a BCA method (Pierce™ BCA Protein Assay Kit, Thermo Fisher Scientific, Lenexa, KS). Equal amounts of protein (35 μg) were loaded and separated by SDS-polyacrylamide gel electrophoresis and transferred onto nitrocellulose (Bio-Rad, CA). Blots were probed using rabbit polyclonal antibodies specific for p-TAK1, p-JNK, p-P38, p-c-Jun, MyD88, total TAK1, TRAF6, O-GlcNAc (CTD110.6), β-actin, and other signaling proteins. The protein bands were visualized by the Bio-Rad Chemidoc system. Blots were stripped and re-probed with β-actin or other protein for equal loading.

### Molecular modeling studies

*Ligand preparation:* The Pentagalloyl glucose or PGG ([(2S,3R,4S,5R,6R)-2,3,5-Tris[(3,4,5-trihydroxybenzoyl)oxy]-6-[(3,4,5-trihydroxybenzoyl)-oxymethyl]oxan-4-yl] 3,4,5-trihydroxybenzoate) ligand has been first optimized by B3LYP/6-311++G** basis set using jaguar8.9 then subjected to the ligand preparation in the LigPrep3.5 of Schrodinger suite 2015.3. *Protein preparation:* The methodology has been adopted from our previous studies to prepare PGG ligand and TAK1, proteins for the docking calculations (17). The three-dimensional structure of TAK1 has been taken from our previous studies. The missing loops and sidechains in the downloaded crystal structures of proteins were modeled, refined, hydrogens added and the protonation state of titratable residues at 7.4 pH was assigned using the protein preparation wizard of Schrodinger suite 2015.3 (31). The non-polar hydrogens were merged and the OPLS2005 force field has been applied. Additional details regarding in silico modeling and docking studies are provided in ***SI Materials and Methods***.

### Rat adjuvant-induced arthritis (AIA) and PGG administration

Female Lewis rats, ~120-150g (Harlan Laboratories, Indianapolis, IN), were injected subcutaneously at the tail base with 300 μL (5 mg/ml) of lyophilized *Mycobacterium butyricum* (Difco Laboratories, Detroit, MI) in sterile mineral oil. The injection of adjuvant was considered day 0. Ankle circumferences and articular index scoring were done from days 0-18 by the blinded observer as described previously (29). The healthy (naïve) rats group served as a control for AIA untreated group. In the treatment group, PGG (25 mg/kg, daily oral gavage) was administered after the appearance of the sign of inflammation (day 9). The Δ ankle circumferences of both the hind ankles from each animal were averaged and ‘n’ is represented as the number of animals used in each of the experimental groups. The proposed animal experiments were approved by the university’s IACUC committee.

### Micro-CT imaging

Joints were harvested and imaged in the Quantum GX (Perkin Elmer). Micro-CT imaging and image analysis with the 3D reconstruction of images using in-built analysis software. The X-ray tube settings were 70 kV and 60 mA to scan in standard mode. The duration of imaging at each time point was 2 mins at FOV 72 mm, and an estimated radiation dose of 12-81mGy. The duration of imaging for Bone Microarchitecture Analysis was set for 14 min scan in high-resolution mode at 90KV/88mA with FOV 36 mm and an estimated radiation dose of 221mGy. The reconstructed images were viewed and analyzed using the Analyze Direct 12.0 software (AnalyzeDirect, Inc., Overland Park, KS).

### Multiplex ELISA

Serum ELISA for multi-panel cytokines was done according to Millipore recommendation (AAH-CYT-5-2). Serum RANKL was determined using the RayBiotech ELISA kit (Cat# ELM-TRANCE-1). Serum was diluted 1:2 for multiplex, while 1:5 for RANKL analysis.

### Regressive hematoxylin and eosin (H&E) staining

Sections were cryosectioned (5 µm) and subsequently fixed in alcoholic formalin. Tissues were then stained with Harris hematoxylin and quickly dipped in acid alcohol. Then sections were dipped in lithium carbonate solution. Sections were rinsed in 95% ethanol and then stained in eosin. Finally, sections were dehydrated, and cover slipped.

### Immunohistochemistry (IHC) staining

Tissue sections were fixed in cold acetone and then treated with 3% peroxidase in 0.1M Tris. Tissues were blocked with 3% horse serum. The sections were incubated with mouse anti-human TAK1, pTAK1, or purified mouse IgG (Coulter) for an additional hour. Then a 1:100 diluted horse anti-mouse biotinylated secondary antibody (Vector) was added to the tissue sections and incubated at room temperature. Then avidin–horseradish peroxidase (BD Pharmingen, San Diego, CA) was added at a 1:10,000 dilution. Finally, diaminobenzidine tetrahydrochloride substrate (DAB) (Vector) was added to the sections. The sections were then counterstained with Harris’s hematoxylin, dipped in saturated lithium carbonate solution for bluing, and then cover slipped.

### Statistical analysis

Statistical analysis was performed using a wat ANOVA test followed by a Tukey’s multiple comparison test to evaluate the statistical significance of group differences in measured parameters from IL-6 and IL-8 protein expression or Western blotting studies in human RASFs. Student’s *t*-test was performed to calculate statistical differences between the means of the different protein variables obtained from *in vivo* findings. *P* values less than 0.05 were considered significant.

## Results

### PGG inhibits multi-cytokine and growth factor expression in IL-1β-induced human RASFs

**Figure 1:**
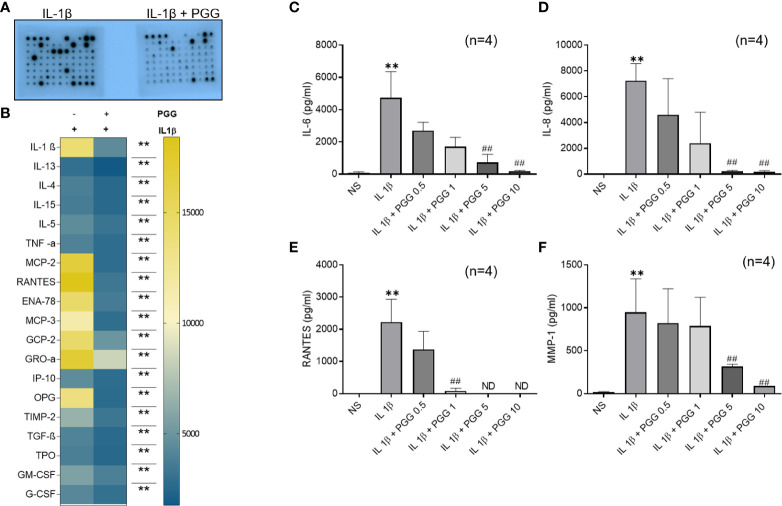
TAK1 regulates IL-1β-induced IL-6 and IL-8 production in RASF. **(A, B)** RASFs were pretreated with PGG (5 µM) for 2 hr, followed by IL-1β (10 ng/ml) stimulation for 24 hours. Cytokines array was done as per instruction and developed on X-ray and analyzed with ChemiDoc™ scanning for intensity **(A, B,** **S1**) IL-6 and IL-8 production was determined in the conditioned media using commercially available ELISA kits. **(C, D)** RANTES and MMP-1 **(E, F)**. The values are represented as mean ± SEM of n=4 experiments using different donors. **p<0.01 for IL-1β *vs* IL-1β+PGG.

Pretreatment of human RASFs with or without PGG (5 μM) for 2 h, followed by IL-1β stimulation resulted in the inhibition of IL-1β (~69%), TNF-α (~30%), chemokines like MCP-1(CCL2), RANTES (CCL5), ENA-78 (CXCL5), MCP-3 (CCL7), GCP-2 (CXCL6) and GRO-α (CXCL1) (~84, 82, 76, 75, 66, and 51%, respectively) while in growth factors ~81% inhibition in OPG and ~51% in TIMP-2 (**Figures 1A, B**, **S1**) when compared to IL-1β (10 ng/ml) stimulated RASFs.

### PGG inhibits IL-1β-induced RASFs IL-6, IL-8, RANTES, and MMP-1 production

IL-1β (10 ng/ml) stimulation resulted in a 160- and 180-fold induction in IL-6 and IL-8 production, respectively (**Figures 1C, D**; p<0.05, p<0.01). Pretreatment with PGG (5 and 10 μM) for 2 h resulted in the inhibition of IL-1β-induced IL-6 (16% and 49%) and IL-8 **(**16% and 49%) production, respectively, when compared to the IL-1β treated samples (p<0.05 at 10 μM). IL-1β stimulation of RASFs resulted in more than 1500- and 50-fold induction in RANTES and MMP-1 levels, respectively (**Figures 1E, F**; p<0.01, p<0.05). IL-1β-induced RANTES/CCL5 was completely inhibited by PGG pretreatment at 5 and 10 μM, while MMP-1 production was inhibited by 16% and 49% when compared to IL-1β stimulated RASFs. The results from this study showed that PGG is effective in suppressing IL-1β-induced inflammatory and tissue destructive factors that are critical mediators in RA pathogenesis.

### O-GlcNAcylation as a posttranslational modification in RA and its inhibition by PGG in IL-1β stimulated RASFs

**Figure 2:**
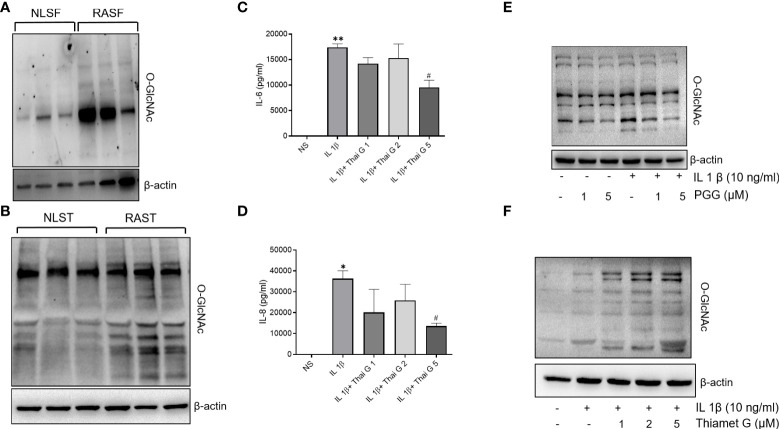
O-GlcNAc activity in NLSF and RASF and selective inhibition by PGG. **(A, B)** O-GlcNAc activity in NLSF, RASF, and normal and RA tissue in different donors. **(C, D)** IL-6 and IL-8 production were determined in the conditioned media using commercially available ELISA kits after overnight pretreatment with Thiamet (1-5 µM) followed by IL-1β stimulation for 24 hours. **(E)** RASFs were pretreated with PGG (1-5 µM) for 2 hour, followed by IL-1β stimulation for 30 minutes. Cell lysates were analyzed for O-GlcNAc and β-actin. **(F)** RASFs were pretreated with PGG (1-5 μM) followed by IL-1β stimulation for 30 minutes. Cell lysates were prepared for analysis of O-GlcNAC and β-actin expression. The values are represented as mean ±SEM of n=4 experiments using different donors. *p<0.05, **p<0.01 for NS vs IL-1β; #p<0.05 IL-1β vs IL-1β+Thiamet. Western immunoblots shown are the representatives of the experiments repeated on 3 or 4 different RASF donors.

Accumulating evidence points to the role of O-GlcNAc as an important PTM in the cytokine signaling network (32, 33). Western blot analysis showed that the O-GlcNAc expression was markedly higher in both human RASFs and RASTs when compared to NLSFs or NLSTs, respectively (**Figures 2A, B**). To understand the involvement of O-GlcNAc in IL-1β signaling, we pretreated RASFs with Thiamet (an inhibitor of O-GlcNAcase; 1-5 μM) overnight followed by IL-1β stimulation of 24 h to determine IL-6 and IL-8 production and O-GlcNAc expression. Pretreatment with Thiamet resulted in a dose-dependent inhibition of IL-1β-induced IL-6 and IL-8 production, which corroborated with the immunoblotting results showing cellular increase in the expression of O-GlcNAc-modified protein by Thiamet in RASFs (**Figures 2C–F**). Overall, these findings suggest O-GlcNAcylation as an important mechanism of posttranslational modification regulating IL-1β-stimulated RASF activation.

### PGG inhibits phosphorylation of TAK1 at Thr^184/187^ and enhances A20 expression in human RASFs

**Figure 3:**
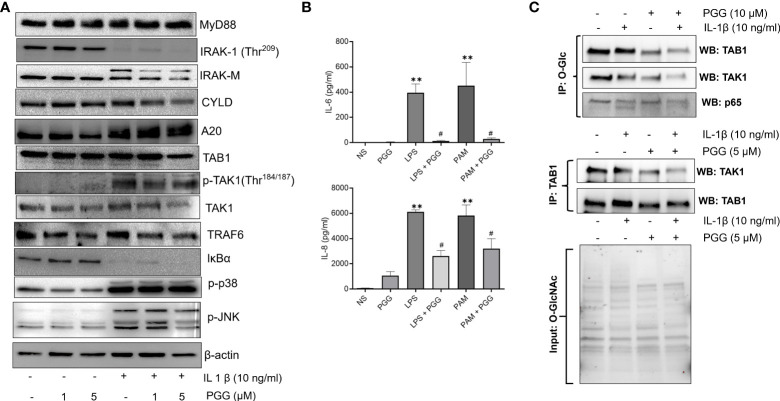
PGG selectively inhibits phosphorylation of TAK1 at the Thr184/187 site to inhibit its kinase activity. **(A)** RASF was pretreated with PGG (1-5 µM) for 2 hr, followed by IL-1β stimulation for 30 minutes. Cell lysates were analyzed for MYD88, IRAK-1, IRAK-M, CYLD, A20, TAB1, pTAK1 (Thr^184/187^), TAK1, TRAF6, I-kBα, p38, pJNK, p-c-Jun, and β-actin. **(B)** PBMC were pretreated with PGG (5 µM), followed by stimulation with a TLR2 agonist (PamCys3, 1 µg/ml), or TLR4 agonist (lipopolysaccharide, LPS; 1 µg/ml), IL-6 and IL-8 IL-6 and IL-8 production was determined in the conditioned media using commercially available ELISA kits. The results in each figure represent the experiments repeated on four RASF from different donors. **(C)** RASFs pretreated with PGG (10 μM) and stimulated with IL-1β, immunoprecipitated (IP) with O-GlcNAc or TAB1, and probe for TAK1, TAB1, and p65, also included input for O-GlcNAc to show equal loading. #p<0.05 IL-1b vs. IL-1b+PGG; **p<0.01 for IL-1β *vs* IL-1β+ PGG.

TAK1 is a central mediator of signal transduction pathways besides other ubiquitin and deubiquitinase in TNF-α or IL-1β-induced MAPK and NF-κB signaling pathways (34). Studies highlight that A20, an ubiquitin-editing enzyme may exert this suppressive effect for NF-κB through the regulation of ubiquitin of signaling molecules with its dual enzymatic activities; ubiquitination and deubiquitination (35) Recent studies suggest that TAK1 phosphorylation at Thr^184/187^ is a critical determinant of its downstream signaling in human RASFs (36, 37). We evaluated the effect of PGG in regulating IL-1β-induced signaling proteins that are proximal the to IL-1R intracellular domain or downstream of TAK1 in the signaling hierarchy in human RASFs (**Figure 3A**). Our results showed that while the pretreatment of PGG (1-5 µM) showed no change in MyD88 expression and was unable to rescue IL-1β–induced IRAK-1 degradation in RASFs, it inhibited IRAK-M phosphorylation in a dose-dependent manner (**Figure 3A**). PGG had no effect on ubiquitin E3 ligase CYLD, but surprisingly increased A20 posttranslational modification indicating its enhanced E3 ligase function or its deubiquitination function with PGG treatment of human RASFs, which suggested its potential regulatory function on IL-1β-induced NF-κB activation. Pretreatment of PGG inhibited RASF pTAK1(Thr^184/187^) expression in a dose-dependent manner without affecting the expression of its associating partners TAB1 or TRAF6, implicating that PGG’s direct interaction with TAK1 is an important mechanism in regulating the IL-1β-activated signaling pathways (**Figure 3A**). Evaluation of the downstream signaling proteins showed that PGG was able to inhibit IL-1β-induced p-JNK, but not p-P38 or the degradation of I-κBα. To further confirm or refute the selectivity of PGG in regulating the IL-1β pathway, we compared the inhibitor potential of PGG against other stimulants such as TLR-2 agonist (Pam3Cys) or TLR-4 agonist (lipopolysaccharide; LPS) in human PBMC. Pretreatment with PGG (5 μM) resulted in the inhibition of TLR-2 and TLR-4-induced IL-6 (96% and 94%) and IL-8 (58% and 48%) production, respectively, when compared to TLR-2 and TLR-4 treated samples (**Figure 3B**; p<0.05 at 5 μM). Overall, these findings suggest that PGG preferentially inhibits IL-1β–induced phosphorylation and activation of TAK1 (Thr^184/187^) to suppress downstream signaling pathways. We further looked at the interacting protein with O-GlcNAc in IL-1β signaling, As in **Figure 3C**, cells were pretreated or untreated and stimulated with IL-1β were IP with O-GlcNAc and probe with TAB1, TAK1 and p65. As shown in Western blot results that TAB1 is heavily glycosylated in RASFs, which further undergo modification with IL-1β treatment shown by the shift of protein band suggesting that IL-1β specifically induces TAB1 glycosylation (**Figure 3C**, upper panel). Importantly, PGG pretreatment reduces both the constitutive and IL-1β-induced O-GlcNAcylation, thereby, potentially impacting the downstream signaling in human RASFs (**Figure 3C** upper panel). In confirmation of this hypothesis, we also observed that PGG reduces the association of TAB1-TAK1 in IL-1β activated RASFs, as shown by IP results with TAK1 and probe for TAK1 expression by Western blot (**Figure 3C**, lower panel). These findings suggest that PGG, by altering the O-GlcNAcylation state of TAB1, reduces the extent of TAK1 activation and its downstream signaling in IL-1β-stimulated RASFs.

### PGG inhibits TAK1 kinase activity by occupying its ATP-binding sites

**Figure 4:**
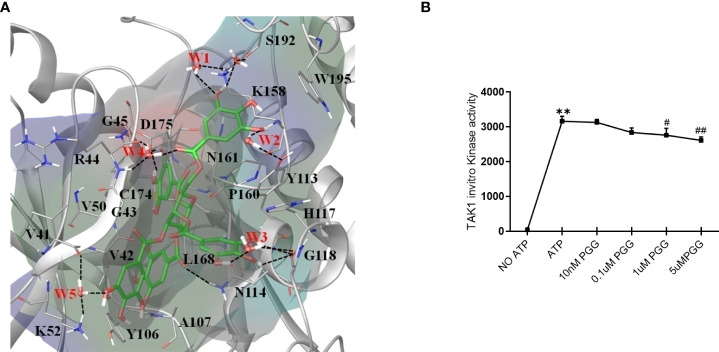
Unique insights from the in silico molecular docking studies of PGG binding in the TAK1-TAB1 complex. **(A)** Molecular dynamics conformation at 50 ns of PGG in the binding site of TAK1-TAB1 complex. Residue properties surface shown in the Red, Blue and Green represent electronegative, electropositive, and hydrophobic surface area of the binding site. The H-bonds are shown as a dotted line. **(B)** Inhibition of the TAK-1 *in vitro* kinase activity by PGG was tested using a kinase assay as per the manufacturer’s instructions. **p<0.01 for No ATP vs ATP. #p<0.05 and ##p<0.01 for ATP vs. PGG.

The non-covalent binding interactions between the PGG ligand and binding site residues forming interactions of all four proteins were assessed through the long molecular docking (MD) simulation. The bonding network of PGG in the binding site of TAK1 involves several direct and indirect (via water) hydrogen bonds with the side chain of residues K52, Y113, N114, K158, D175, S192, and the backbone of residues G45, R44, V41, G118 (**Figure 4A**). Furthermore, trihydroxy benzoyl rings of PGG are perfectly positioned at the inner and outer binding sites to establish π-π interactions with Y106, Y113, H117, and W195, which play a very important role in the structural stability of the ligand (38). The residue properties surface was generated to visualize the chemical nature of the binding cavity, have shown that hydrophobic residues are significantly present in the binding cavity which contributes to the stability of the ligand. The binding site nature and protein-ligand interactions viz. H-Bonds, hydrophobic, and water bridges throughout the simulation suggest a stronger preference for the TAK1 (see supporting information **Figures S1–S6**). The PGG ligand has been drawn away from the TRAF6 binding site during the early MD simulation whereas it was loosely bound to the IRAK1 and IRAK4 throughout the trajectories which suggest that PGG may not be the ligand of these proteins. For further insights into PGG inhibition of TAK1 kinase activity, we performed *in vitro* kinase assay, PGG showed dose-dependent inhibition of TAK1 activity (**Figure 4B**).

### Oral administration of PGG ameliorates rat AIA and reduces bone damage

**Figure 5:**
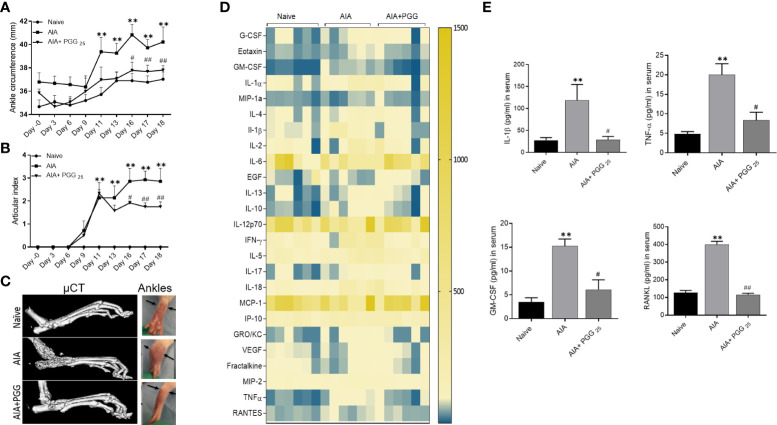
PGG modulates the in vivo inflammatory cytokines to inhibit rat AIA. PGG (25 mg/kg/day, oral) administration for 10 days (starting at day 8) ameliorated rat AIA as evident from reduced ankle circumferences and paw scoring **(A, B)**. The ankle circumferences of both the hind ankles from each animal were averaged and ‘n’ is represented as the number of animals used in each of the experimental groups. **(C)** Joints from naive, AIA, and PGG treated were analyzed in MicroCT for bone damage, and pictures were taken from each group. **(D, E)** Serum isolated from animals were used in Multiplex ELISA for 27 analytes (Heatmap) and RANKL ELISA as per the manufacturer’s instructions. The results in each figure represent the experiments repeated on 6 animals from two different studies. **p<0.01 vs naïve group; #p<0.05, ##p<0.01 vs AIA group.

Clinical signs of the AIA indicating swelling and erythema of one or more ankle joints followed by involvement of the metatarsal and interphalangeal joints, first appeared in the hind paws between 8 and 9 days after immunization, with >95% incidence around day 13. Administration of daily oral gavage of PGG (25 mg/ml) significantly suppressed the clinical severity and progression of AIA in treated rats (**Figures 5A, B**; p<0.01). PGG administration to immunized rats reduced the progression of arthritis evidenced by inhibition in arthritis score compared to RA rats. The joint destruction in naive, AIA and AIA+PGG treated groups was assessed using micro-CT imaging (**Figure 5C**). We observed severe bone erosion in untreated AIA rats when compared to ankles from naïve rats, which displayed smooth cartilage and intact bone surface.

### PGG preferentially inhibits IL-1 and RANKL *in vivo*

To correlate the efficacy of PGG in regulating the clinical severity of rat AIA with the biochemical and histological changes, we looked at the serum levels of cytokines, chemokines, and growth factors using a multiplex or RANKL ELISA kit. Our results showed among 27 analytes measured, PGG administration markedly reduced the levels of IL-1β, TNF-α, GM-CSF, and RANKL (**Figures 5D** **, E**; p<0.05, p<0.01). These results suggest that PGG preferentially inhibits IL-1 family proteins to limit inflammation and tissue destruction in RA. These findings further validate the amelioration of the disease in rat AIA and restoration of bone integrity as observed by micro-CT analysis.

### PGG modulates *in vivo* phosphorylation of TAK1 to ameliorate rat AIA

**Figure 6:**
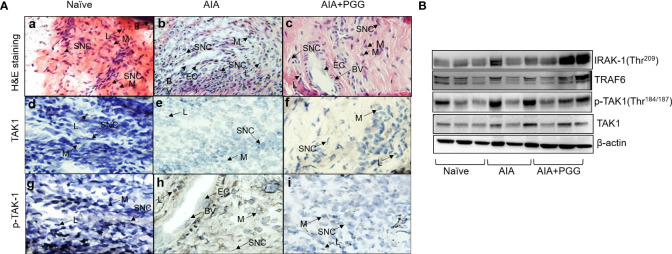
PGG modulates phosphorylation of TAK-1 activity *in vivo*. (A) H&E staining of rat ankles obtained from normal rats, rats given AIA, and rats were given AIA and Penta-O-galloyl-β-D-glucose (PGG) shows inflammation. In addition, pTAK1 and TAK1 were immunolocalized *via* IHC staining. **(A)** Naïve rat ankle stained *via* H&E shows no inflammation and synoviocytes (SNC), lymphocytes (L), bone (B), and macrophages (M) are present. **(B)** AIA rat ankle stained by H&E shows high amounts of inflammation including the presence of endothelial cells (EC) which comprise blood vessels (BV) and many synoviocytes (SNC), lymphocytes (L), and macrophages (M). **(C)** AIA+ PGG rat ankles stained by H&E shows low levels of inflammation and that endothelial cells (EC), blood vessels (BV), synoviocytes (SNC), lymphocytes (L), and macrophages (M) are present. **(D)** There is no staining for TAK1 in naïve rat ankles. **(E)** There is minimal staining in synoviocytes (SNC) in AIA rat ankles. **(F)** There is minimal staining in synoviocytes (SNC) in AIA+PGG rat ankles. **(G)** There is no staining for p-TAK-1 in naïve rat ankles. **(H)** There is intense staining for p-TAK-1 in macrophages (M), synoviocytes (SNC), and lymphocytes (L). **(I)** There is a minimal staining in macrophages (M), synoviocytes (SNC), and lymphocytes (L). NOTE: the same tissue of each treatment was incubated with nonspecific IgG, which showed no expression. (Original magnification × 40). (B) Joint homogenates (30 µg per sample) from naïve, AIA, and PGG treated rats were analyzed for the expression of IRAK-1 (Thr^209^), pTAK1 (Thr^184/187^), TAK1, TRAF6, and β-actin.

To further unearth the potential molecular targeting of TAK1 by PGG, we performed Western blot analysis on the joint homogenates in conjunction also H&E and immunohistochemistry for TAK1 and p-TAK1 in the sectioned ankles. The H&E staining indicated a higher degree of infiltrating cells in AIA joints that was markedly reduced by PGG administration (**Figures 6A** **, B**) the inflammation presents in each treatment group. As shown, the AIA group exhibited profound inflammation as compared to the naïve group. In addition, those treated with both AIA and PGG appear to have less inflammation than the AIA group as shown in **Figures 6B, C**. **Figures 6D–F** shows TAK1 expression in rat ankles in naïve, AIA, and AIA+PGG groups, respectively. In the AIA and AIA+PGG treated groups, TAK1 is expressed mostly in synoviocytes as seen in **Figures 6E, F**. Also, there is no change in the staining of TAK1 between these two treatments. However, p-TAK1 expression was much higher in the AIA rat ankles than in the naïve as shown in **Figures 6G, H**; p-TAK1 appears to be expressed on lymphocytes, macrophages, and synoviocytes as shown in **Figure 6H**. In addition, p-TAK1 expression in these cells is higher in the AIA compared to the PGG treated AIA rat ankles seen in **Figures 6H, I**. These results were confirmed with Western blot analysis of the joint homogenates that showed an increase in p-TAK-1 expression in the AIA group compared to the naïve group, which was inhibited with PGG treatment (**Figure 6B**). However, no marked difference in TAK1 or TRAF6 expression in the joint homogenates of AIA or PGG-treated rats was observed. These findings indicate that PGG ameliorates AIA by downregulating TAK1.

## Discussion

In the present study, we provided evidence that GlcNAcylation plays an important role in pro-inflammatory cytokine signaling networks mediated through TAK1 in human RASFs. Our study identifies a novel anti-arthritic property of the natural compound PGG using IL-1β-induced signaling pathways in human RASFs and the rat AIA model of human RA. Furthermore, we provide evidence that PGG reduces TAK1 activation, primarily by down-regulating the GlcNAcylation of its structural partner TAB1, thereby interfering with downstream signaling activation by IL-1β. These findings suggest that TAK1 mediated signal transduction pathways are important therapeutic target in cytokine signaling networks and PGG or its structurally similar molecules may be developed for further pre-clinical testing in the treatment of RA.

While the process of O-GlcNAcylation was first identified in 1983, its role has been explored in the last two decades with the studies showing its role in chronic ailments such as type II diabetes, Alzheimer’s disease, and tumor malignancy (11, 39). Regarding rheumatic diseases, the role of GlcNAcylation is an understudied area of investigation. A recent study by Kim et al., suggests that O-GlcNAcylation of NF-κBp65 upon TNF-α stimulation is a key mechanism of its nuclear translocation in RASFs (7). Another study points to the increased expression of O-GlcNAc modifications in the cartilage of OA patients (9). However, no study so far has provided evidence for its role in facilitating inflammation and tissue destruction mediated by RASFs. Our findings not only underline the importance of this PTM in cytokine signaling in RASFs but also provides evidence that PGG, a natural compound, can inhibit IL-1β-induced inflammation and tissue destruction, partly, by down-regulating O-GlcNAc modifications of several key signaling proteins, thereby, limiting their role in activating inflammatory genes.

The binding of IL-1 to the IL-1R complex recruits an adaptor protein (MyD88) and consequentially initiating phosphorylation of IL-1 receptor-associated kinases (IRAKs) (40). Phosphorylated IRAK-1 facilitates TRAF6 recruitment to the complex and subsequent IRAK-1 degradation (41). IL-1 triggers activation of TAK1 by the Lys^63^-linked poly-ubiquitination of TRAF6, which binds to the C-terminal zinc-finger motifs of TAB2 and TAB3, inducing autophosphorylation and activation of TAK1 (42). TAK1 eventually phosphorylates MAPK and IKK, resulting in nuclear translocation of the transcription factors activation protein-1 (AP-1) and NF-κB. Pathak et al. showed that TAB1 O-GlcNAcylation enhances I-κBα phosphorylation, which in turn regulates NF-κB activation (43). Though PGG pretreatment was incompetent in blocking IRAK-1 degradation, however, it markedly inhibited *in vitro* TAK1 kinase activity. Molecular docking studies using TAK-1 and TAB1 fusion protein homology structural modeling provides supporting evidence that PGG can bind residues near ATP-binding sites resulting in the blockade of its kinase activity. However, our study points to the fact that the inhibition of TAB1 O-GlcNAcylation by PGG that impacted its TAK1 activation and suppression of inflammatory mediators produced by activated RASFs.

Most notably, this study provides mechanistic insights into the GlcNAcylation that governs TAK1 activation through TAB1 in RASF and an underlying mechanism of PGG interaction with IRAK-1/TRAF6/TAK1 that results in the inhibition of TAK1 activity. These findings suggest that testing O-GlcNAc inhibitors to effectively disrupt the integrated cytokine signaling will limit their role in RA pathogenesis. TAK1 is essential in several cytokine-mediated signal transduction cascades, including the TNF-α, IL-1β, and TGF-β pathways, as well as signaling downstream of TLR and NOD1/2 (44). Its native forms comprise of catalytic kinase subunit in complex with a regulatory subunit TAB1, and either of two homologous proteins, TAB2 or TAB3 (45). Our results in IL-1β-activated RASFs showed no change in MyD88, which is an adaptor protein function to pass the signal to IRAK which degrades in IL-1β stimulation and is unable to recover even after treatment with PGG. Studies with Tab1-deficient mouse embryonic fibroblasts suggest that TAB1 is essential in the regulation of the TAK1 complex and inducing its catalytic activity (46). Pathak et al. showed that O-GlcNAcylation of a residue (Ser^395^) on TAB1 modulates TAK1 activation in response to IL-1β stimulation or osmotic stress (43). O-GlcNAcylation of TAB1 induces a significant increase in TAK1 autophosphorylation and its activation, which eventually phosphorylates and activates downstream MAPK and IKK. While other TAB partners of TAK1, namely TAB2 or TAB3, may be involved in the regulation of TAK1 activity in other chronic conditions such as cancer (47, 48), our focus remains on TAK1/TAB1 complex given its established role in IL-1β-induced TAK1 activation in human RASFs (17).

**Figure 7:**
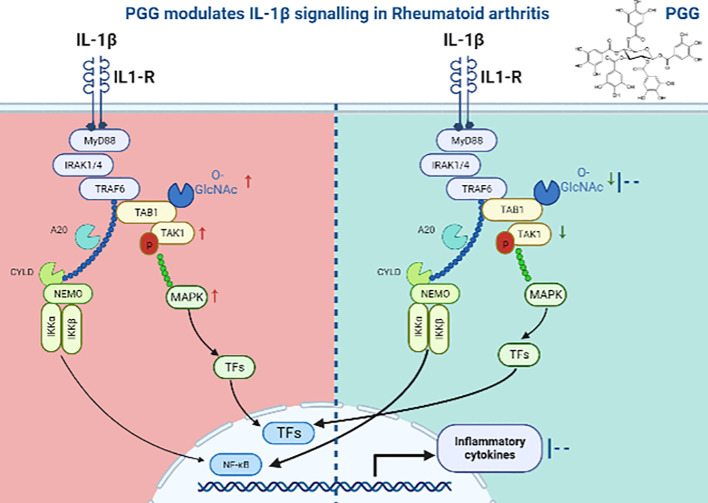
Schematic figure showing the mechanism of PGG in IL1β signaling. In IL1β signaling, IL-1β activates the signal through its receptor to MyD88, an adaptor protein which further recruits IRAK1-4 and activates TAK1 dependent inflammation. We showed that PGG suppresses IL-1β-induced post translation modification (O-GlcNAC) and downstream inflammatory responses by inhibiting multiple cytokines.

The currently available therapies for RA are expensive, immunosuppressive, and even in some patients, they are unresponsive which is attributed to our restricted understanding of the development of RA. These therapies are sometimes unsuitable for long-term use (49, 50). Additionally, the existing RA therapies are B-cell-, T-cell-, and cytokine-directed, and no existing medication for RA directly targets SFs despite an established body of molecular and clinical evidence suggesting their role in RA pathogenesis (51–53). While the central role of TAK1 is well established in TNF-α, IL-1β, or TLR signaling, recent findings from our lab highlighted a novel role of TAK1 in chronic IL-6 trans-signaling and RANKL-driven signaling in human RASFs (54). As summarized in the graphical abstract (**Figure 7**), we provide another evidence for targeting TAK1 for the amelioration of RA and describe a unique mechanism through which PGG inhibits the interaction between TAK1 and its associated signaling molecules which are important in cytokine signaling in inhibiting inflammation and tissue destruction in RA.

## Data availability statement

The raw data supporting the conclusions of this article will be made available by the authors, without undue reservation.

## Ethics statement

The animal study was reviewed and approved by Washington State University IACUC Committee.

## Author contributions

SU and AKS performed experimental studies and analyzed the data. MC performed MD simulation studies and analyzed the data and prepared the resulting figures. SMR and JHR performed and analyzed histological and IHC experiments. SA conceived the study idea, directed SU and AKS for the *in vitro* and animal studies, and provided the funding support for the study. SU and SA drafted the manuscript. All authors have reviewed the final draft.

## Funding

This study was supported by the NIH grants AR063104 and AR072615 (S.A.), and the funds from Washington State University.

## Acknowledgments

The authors thank lab members for their critical comments and support in animal studies.

## Conflict of interest

The authors declare that the research was conducted in the absence of any commercial or financial relationships that could be construed as a potential conflict of interest.

## Publisher’s note

All claims expressed in this article are solely those of the authors and do not necessarily represent those of their affiliated organizations, or those of the publisher, the editors and the reviewers. Any product that may be evaluated in this article, or claim that may be made by its manufacturer, is not guaranteed or endorsed by the publisher.
