# Supplementary material for: Penta-o-galloyl-beta-d-Glucose (PGG) inhibits inflammation in human rheumatoid arthritis synovial fibroblasts and rat adjuvant-induced arthritis model
Source: Front Immunol. 2022 Aug 10;13:928436. doi: 10.3389/fimmu.2022.928436 (PMC9400595; doi:10.3389/fimmu.2022.928436)
Supplement: Supplementary file 1 [file DataSheet_1.pdf]

Supplementary Material

Supplementary Materials and Methods

Molecular Modelling Studies:

Ligand preparation: The Pentagalloyl glucose or PGG ([(2S,3R,4S,5R,6R)-2,3,5-Tris[(3,4,5-trihydroxybenzoyl)oxy]-6-[(3,4,5-trihydroxybenzoyl)oxymethyl]oxan-4-yl] 3,4,5-trihydroxybenzoate) ligand has been first optimized by B3LYP/6-311++G** basis set using jaguar8.9 then subjected to the ligand preparation in the LigPrep3.5 of Schrodinger suit 2015.3 [1,2].

Protein preparation: The methodology has been adopted from our previous studies to prepare PGG ligand and TAK1 protein for the docking calculations [3]. The missing loops and side-chains in the downloaded crystal structures of proteins were modeled, refined, hydrogens added and the protonation state of titratable residues at 7.4 *p*H was assigned using the protein preparation wizard of Schrodinger suite 2015.3 [4]. The non-polar hydrogens were merged and the OPLS2005 force field has been applied.

Docking: The prepared protein structures of TAK1 was subjected to docking. The 20Å size grid has been generated from the binding site center of all four proteins then the PGG ligand was docked in all proteins in the predefined grid using the rigid docking protocol of GLIDE6.8 [5]. The docking has been performed first using the default setting of standard precision (SP) then the conformations obtained from SP were used as input for extra precision (XP). The 10 best conformations of the ligand were generated. The selection of the best pose has been made on the basis of energy, cluster RMSD and interaction with the respective protein.

Molecular Dynamics: In order to take into account the protein flexibility and evaluate the dynamic stability of the predicted protein-ligand interactions, the best ligand pose complexes obtained from docking were submitted to molecular dynamics (MD) simulations. All docked ligand-protein complexes were solvated in an orthorhombic TIP3P water box and simulations were done in Desmond4.3 using the OPLS 2005 force field [6,7]. The counter ions have been added to neutralize the systems. The complete system generated in this manner consists of about 50,000 atoms. The systems were initially relaxed without any substantial deviation from the initial structure using the default protocol of Desmond. The equilibrated systems were subjected to the final production run of 50 ns with a 2.0 fs time step for bonding, van der Waals, and short-range Coulomb interactions and a 6.0 fs time step for long-range Coulomb interactions during which isothermic isobaric ensemble (NPT) were employed to maintain the constant pressure (1 bar) and temperature (300 K) using Berendsen barostat and thermostat algorithms respectively. The frames in the trajectory were recorded at an interval of 10 ps to study the conformational variations upon ligand binding.

References:

1. Bochevarov, A.D.; Harder, E.; Hughes, T.F.; Greenwood, J.R.; Braden, D.A.; Philipp, D.M.; Rinaldo, D.; Halls, M.D.; Zhang, J.; Friesner, R.A., "Jaguar: A high-performance quantum

chemistry software program with strengths in life and materials sciences," Int. J. Quantum Chem., 2013, 113(18), 2110-2142

2. Schrödinger Release 2015-3: LigPrep, version 3.5, Schrödinger, LLC, New York, NY, 2015.
3. AK Singh, S Umar, S Riegsecker, M Chourasia, S Ahmed. Regulation of Transforming Growth Factor beta-Activated Kinase 1 Activation by Epigallocatechin-3-Gallate in Rheumatoid Arthritis Synovial Fibroblasts Suppression of K-63-Linked Autoubiquitination of Tumor Necrosis Factor Receptor-Associated Factor 6. Arthritis Rheumatol. 68 (2), 347-358
4. Sastry, G.M.; Adzhigirey, M.; Day, T.; Annabhimoju, R.; Sherman, W., "Protein and ligand preparation: Parameters, protocols, and influence on virtual screening enrichments," J. Comput. Aid. Mol. Des., 2013, 27(3), 221-234
5. Friesner, R.A.; Murphy, R.B.; Repasky, M.P.; Frye, L.L.; Greenwood, J.R.; Halgren, T.A.; Sanschagrin, P.C.; Mainz, D.T., "Extra Precision Glide: Docking and Scoring Incorporating a Model of Hydrophobic Enclosure for Protein-Ligand Complexes," J. Med. Chem., 2006, 49, 6177–6196
6. Jorgensen WL, Chandrasekhar J, Madura JD, Impey RW, Klein ML (1983) Comparison of simple potential functions for simulating liquid water. J Chem Phys 79: 926–935.
7. Kaminski GA, Friesner RA, Tirado RJ, Jorgensen WL (2001) Evaluation and reparametrization of the OPLS-AA force field for proteins via comparison with accurate quantum chemical calculations on peptides. J Phys Chem B 105: 6474–6487.

Supplementary Table 1

Chemokines	IL-1 β	IL-1 β + PGG	% decrease
MCP-2	16743.9	2755.2	83.5
RANTES	18411.8	3354.7	81.8
ENA-78	14593.6	3572.1	75.5
MCP-3	11375.8	2838.9	75
GCP-2	14628.3	4975.6	66
GRO-α	17013.7	8423.1	50.5
IP-10	4468.8	2761.6	38.2
TARC (CCL17)	4385.2	3143.2	28.3
MIP-1δ	2894.4	2087.4	27.9
MIP-3 α	3821.6	2769.1	27.5
I-309	2601.2	1938.9	25.5
GRO	19936.3	14876.6	25.4
MCP-1	15677.8	12077.1	23
SDF-1α	3625	2801.1	22.7
FRACTALKINE	4234.5	3324	21.5
MCP-4	3594.5	2883.2	19.8
BLC	4040.7	3297.2	18.4
MIP-1β	5393.7	4434.7	17.8
NAP-2	3956	3332.3	15.8
CK -β 8-1	3750.1	3173.5	15.4
EOTAXIN-2	3545.4	3245.6	8.5
IL-8	17457.8	16415.1	6
MIG	4215.4	4021.8	4.6
EOTAXIN-3	3205	3071.6	4.2
MDC	4080.3	3911.5	4.1
EOTAXIN-01	3486.1	3459.9	0.8
**Growth factors and others**	**IL-1 β**	**IL-1 β + PGG**	**% decrease**
OPG	13492.7	2647.6	80.4
TIMP-2	6570.3	3281.5	50.1
TGF-β	3968.8	2618.3	34
TPO	3776.9	2518.2	33.3
GM-CSF	5636.6	3830	32.1
G-CSF	4225.3	2987.8	29.3
LEPTIN	3478.7	2527.4	27.3
BDNF	4885.2	3552.3	27.3
VEGF-A	3427.8	2517.7	26.6
ANGIOGENIN	4298	3198.3	25.6
OPN	3184.9	2460.2	22.8
IGFBP-4	2565.7	2019.9	21.3
FGF-7	2502	1982.5	20.8
MIF	4663.8	3757.5	19.4

FGF-4	2650.9	2136.7	19.4
PDGF-BB	3022.1	2487.5	17.7
FGF-6	2620.7	2158.7	17.6
FLT-3 LIGAND	3085.5	2550.5	17.3
M-CSF	5467	4564.6	16.5
GDNF	3894.1	3256	16.4
NT-4	2454.5	2079.3	15.3
PARC	3509	3022.4	13.9
FGF-9	3554.4	3108.3	12.6
PLGF	3034.4	2661.1	12.3
IGFBP-3	2155	1958.9	9.1
HGF	2905.8	2654.6	8.6
TGF β-2	3864.5	3563.6	7.8
TIMP-1	13781.5	12781.3	7.3
TGF β-3	3254.6	3065.2	5.8
IGFBP-2	3851.9	3677.3	4.5
NT-3	3854.9	3774.5	2.1
IGFBP-1	3288.4	3221.1	2
IGF-1	4069.4	4062.5	0.2
EGF	3795.8	3911.5	-3

**Cytokines**	**IL-1 β**	**IL-1 β + PGG**	**% decrease**
IL-1 β	14045.5	4450.9	68.3
IL-13	2932.7	1769.8	39.7
IL-4	3789.5	2552.3	32.7
IL-15	3673.5	2550.9	30.6
IL-5	4499.3	3166	29.6
TNF -α	3953.3	2794.6	29.3
SCF	3709.7	2628.8	29.1
OSM	3750.2	2751	26.6
IL-1 α	3005	2285.4	23.9
IL-16	3097.8	2356.5	23.9
IL-3	3916.4	3017.8	22.9
IL-2	3811.6	3019.3	20.8
IFN-γ	3905.6	3107.6	20.4
IL-7	4505.8	3855.2	14.4
TNFSF 14 (LIGHT)	3577.5	3080.9	13.9
IL-12	2811	2452.3	12.8
IL-10	4114	3607	12.3
LIF	3404.2	2986.8	12.3
IL-6	20983.6	19691.4	6.2
TNF-β	4053.5	3887.6	4.1

Supplementary Figures S1-S6

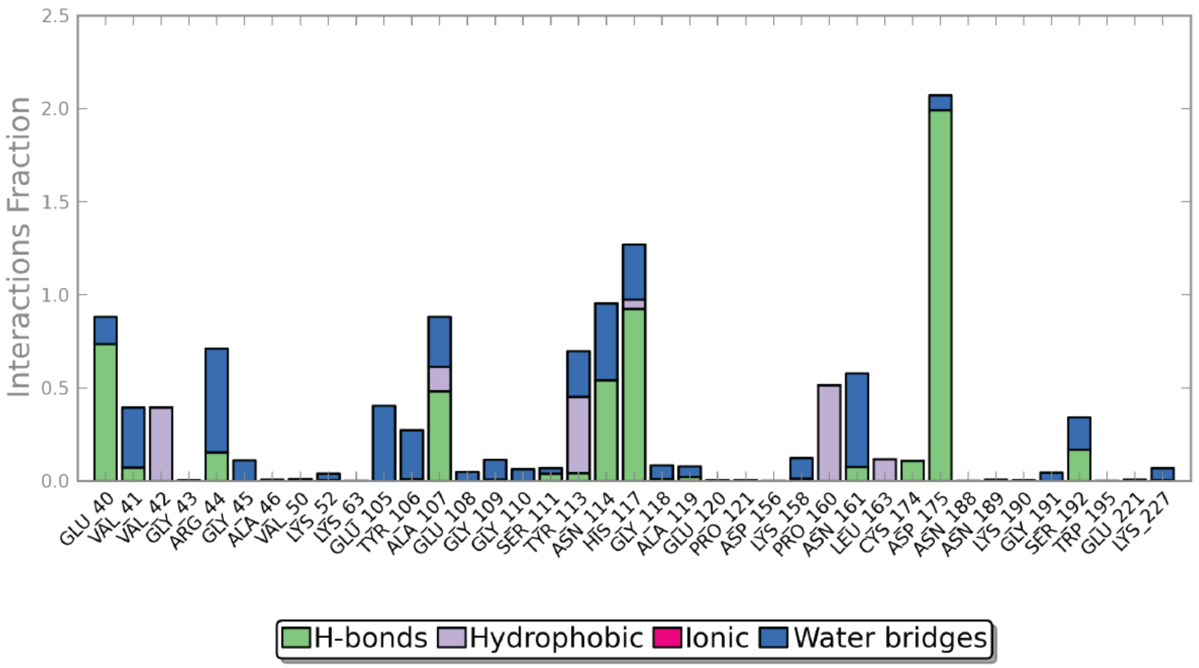

Figure S1. TAK1-PGG complex; Protein-ligand interactions viz. Hydrogen Bonds, Hydrophobic, Ionic and Water Bridges have been shown throughout the simulation. The stacked bar charts are normalized over the course of the trajectory: for example, a value of 0.5 suggests that 50% of the simulation time the specific interaction is maintained. Values over 1.0 means that some protein residue may make multiple contacts of the same subtype with the ligand. Water Bridges are hydrogen-bonded protein-ligand interactions mediated by a water molecule.

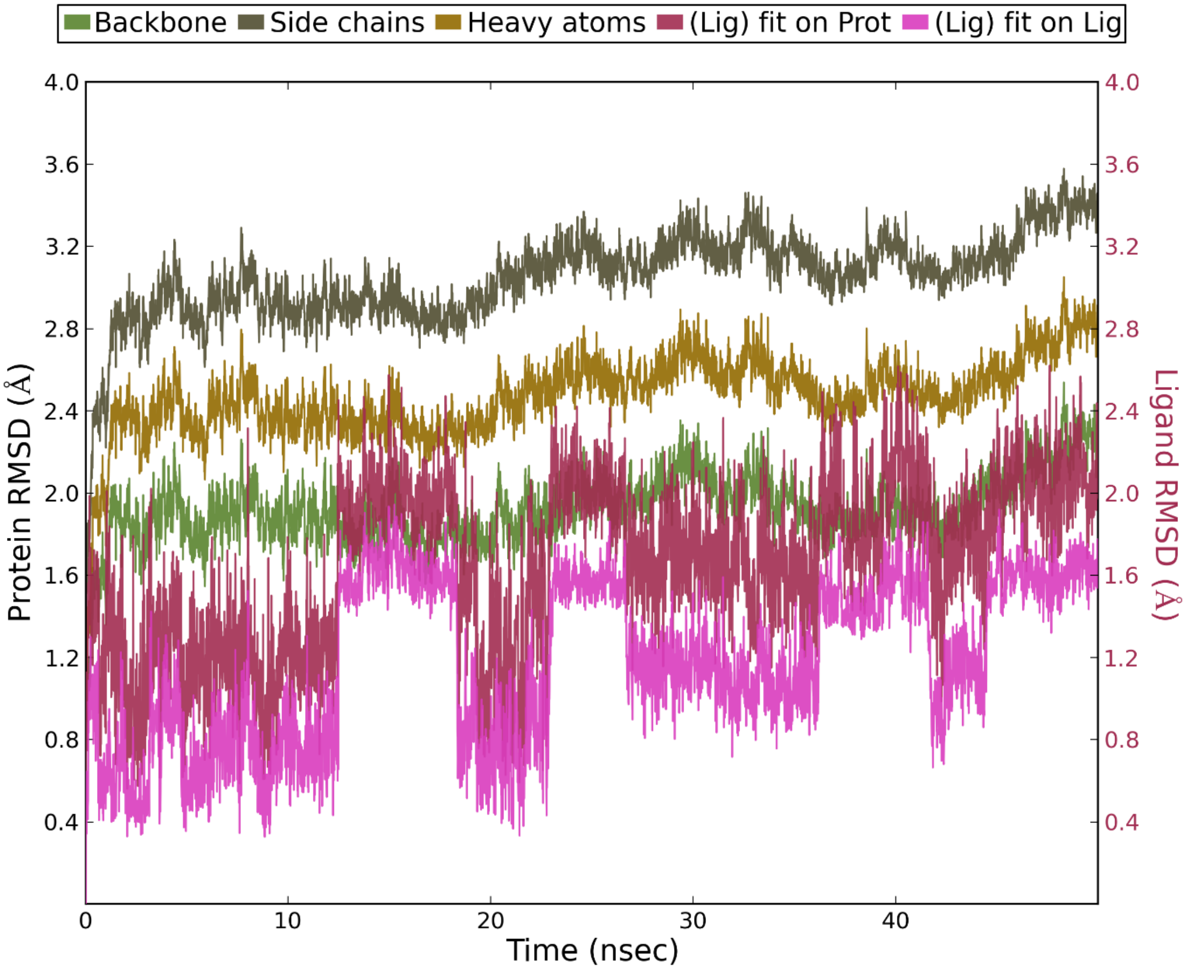

Figure S2. TAK1-PGG complex; The above plot shows the RMSD evolution of a protein (left Y-axis) and ligand (right Y-axis). All protein frames are first aligned on the reference frame backbone, and then the RMSD is calculated based on the atom selection like backbone, side chains, heavy atoms, ligand fit on protein and ligand fit on ligand. RMSD plots indicate that the simulation has equilibrated well. 'Lig fit Prot' shows the RMSD of a ligand when the protein-ligand complex is first aligned on the protein backbone of the reference and then the RMSD of the ligand heavy atoms is measured. This RMSD measures the ligand diffusion in the protein from its initial binding site. 'Lig fit Lig' shows the RMSD of a ligand that is aligned and measured just on its reference conformation. This RMSD value measures the internal fluctuations of the ligand atoms.

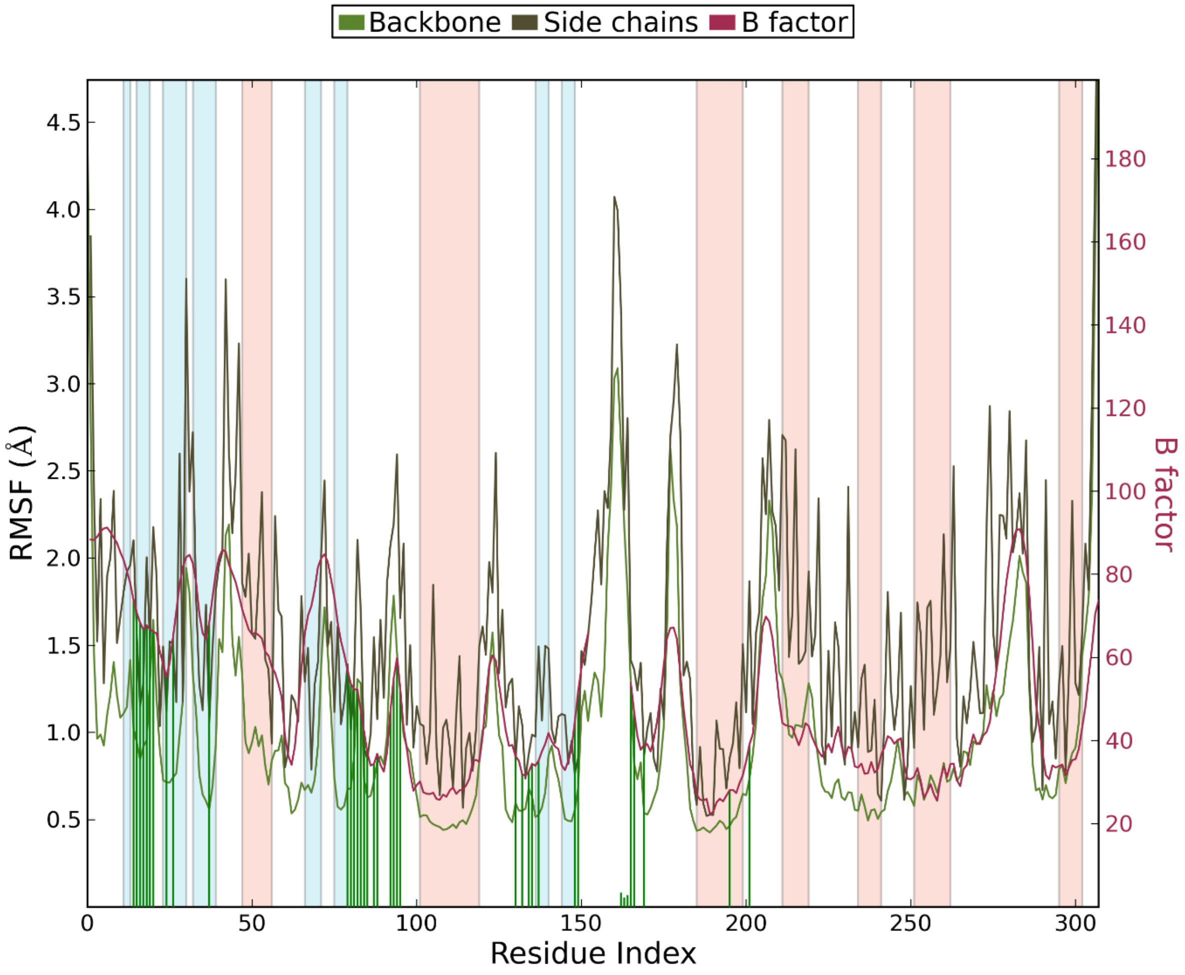

Figure S3. TAK1-PGG complex; In the RMSF plot, peaks indicate residue fluctuations of the protein during the simulation. The N- and C-terminal along with the loop regions of the CB2 fluctuate more than the TM domain of the protein. α-helices are highlighted in pink background. Protein residues that interact with the ligand are marked with green-colored vertical bars.

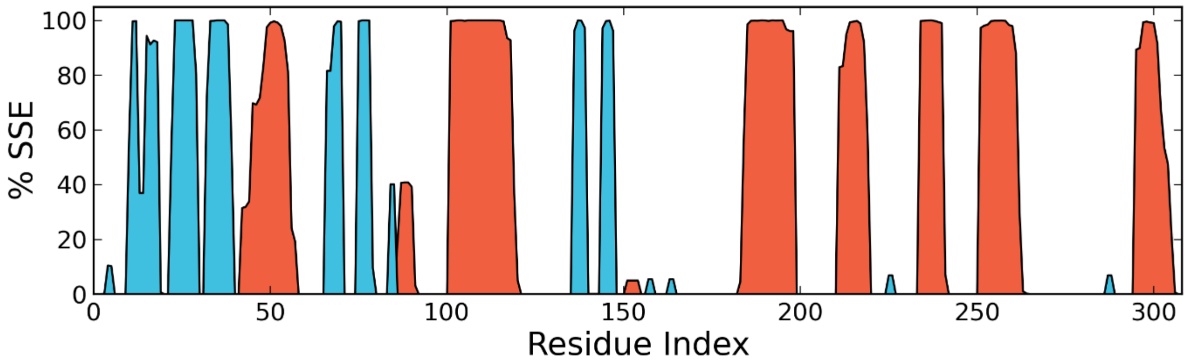

Figure S4. TAK1-PGG complex; The plot above reports Protein secondary structure elements (SSE) throughout the protein structure. White: loop, Red: Helix, Blue: Strand, Grey: Turn.

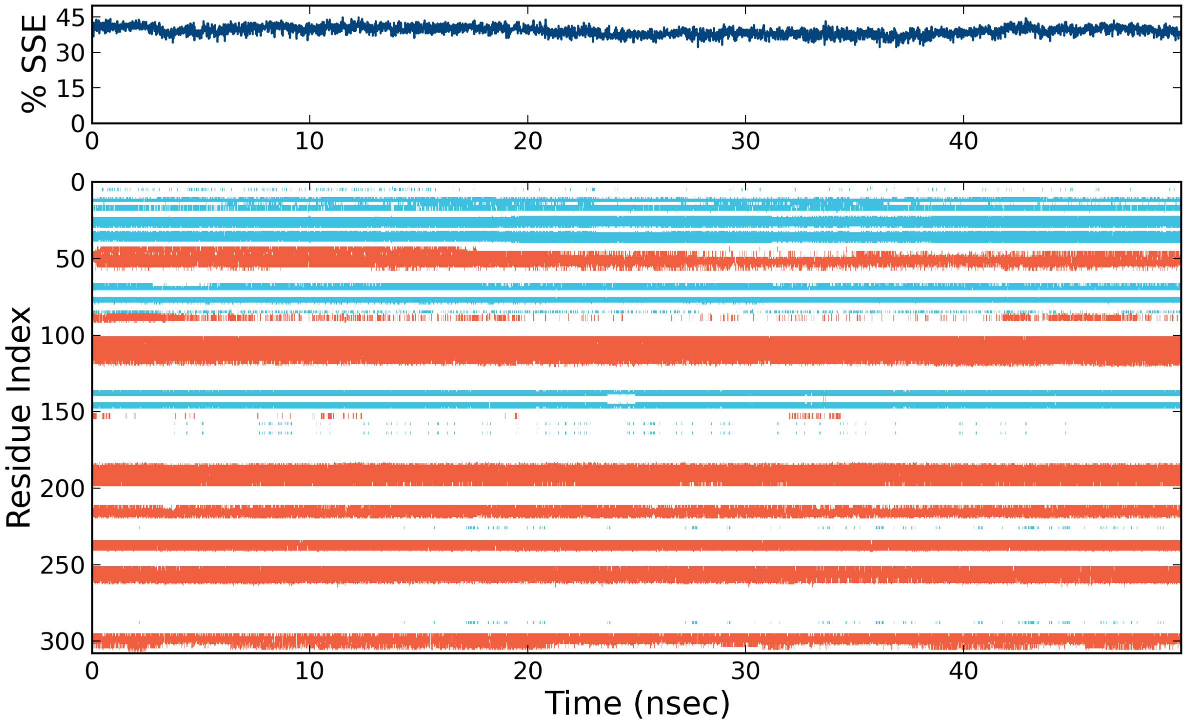

Figure S5. TAK1-PGG complex; The plot above summarizes the Protein secondary structure elements (SSE) composition for each trajectory frame over the course of the simulation, and the plot at the below monitors each residue and its SSE assignment over time. White: loop, Red: Helix, Blue: Strand, Grey: Turn.

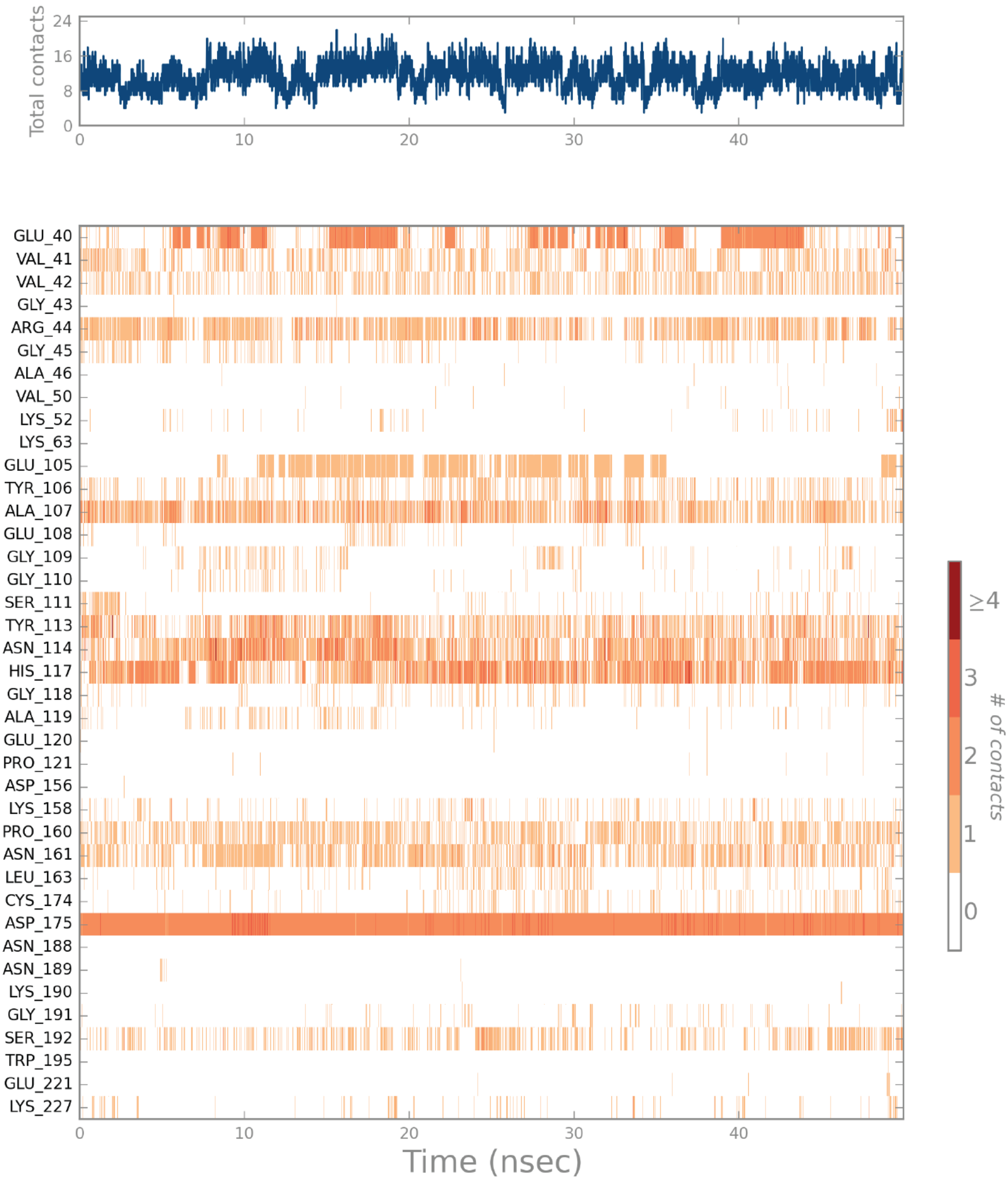

Figure S6. TAK1-PGG complex; The total number of specific contacts make between the protein-ligand complex over the course of the trajectory is shown in the top panel while the residues which interact with the ligand in each trajectory frame is shown in the bottom panel.
